# Recruitment of Patients With Cancer for a Clinical Trial Evaluating a Web-Based Psycho-Oncological Intervention: Secondary Analysis of a Diversified Recruitment Strategy in a Randomized Controlled Trial

**DOI:** 10.2196/42123

**Published:** 2023-11-27

**Authors:** Angeliki Tsiouris, Anna Mayer, Jörg Wiltink, Christian Ruckes, Manfred E Beutel, Rüdiger Zwerenz

**Affiliations:** 1 Department of Psychosomatic Medicine and Psychotherapy University Medical Center of the Johannes Gutenberg-University Mainz Mainz Germany; 2 Interdisciplinary Center for Clinical Trials University Medical Center of the Johannes Gutenberg-University Mainz Mainz Germany

**Keywords:** psycho-oncology, cancer, recruitment, social media, web-based interventions, web-based recruitment

## Abstract

**Background:**

Participant recruitment poses challenges in psycho-oncological intervention research, such as psycho-oncological web-based intervention studies. Strict consecutive recruitment in clinical settings provides important methodological benefits but is often associated with low response rates and reduced practicability and ecological validity. In addition to preexisting recruitment barriers, the protective measures owing to the COVID-19 pandemic restricted recruitment activities in the clinical setting since March 2020.

**Objective:**

This study aims to outline the recruitment strategy for a randomized controlled trial evaluating the unguided emotion-based psycho-oncological online self-help (*epos*), which combined traditional and web-based recruitment.

**Methods:**

We developed a combined recruitment strategy including traditional (eg, recruitment in clinics, medical practices, cancer counseling centers, and newspapers) and web-based recruitment (Instagram, Facebook, and web pages). Recruitment was conducted between May 2020 and September 2021. Eligible participants for this study were adult patients with any type of cancer who were currently receiving treatment or in posttreatment care. They were also required to have a good command of the German language and access to a device suitable for web-based interventions, such as a laptop or computer.

**Results:**

We analyzed data from 304 participants who were enrolled in a 17-month recruitment period using various recruitment strategies. Web-based and traditional recruitment strategies led to comparable numbers of participants (151/304, 49.7% vs 153/304, 50.3%). However, web-based recruitment required much less effort. Regardless of the recruitment strategy, the total sample did not accurately represent patients with cancer currently undergoing treatment for major types of cancer in terms of various sociodemographic characteristics, including but not limited to sex and age. However, among the web-recruited study participants, the proportion of female participants was even higher (*P*<.001), the mean age was lower (*P*=.005), private internet use was higher (on weekdays: *P*=.007; on weekends: *P*=.02), and the number of those who were currently under treatment was higher (*P*=.048). Other demographic and medical characteristics revealed no significant differences between the groups. The majority of participants registered as self-referred (236/296, 79.7%) instead of having followed the recommendation of or study invitation from a health care professional.

**Conclusions:**

The combined recruitment strategy helped overcome general and COVID-19–specific recruitment barriers and provided the targeted participant number. Social media recruitment was the most efficient individual recruitment strategy for participant enrollment. Differences in some demographic and medical characteristics emerged, which should be considered in future analyses. Implications and recommendations for social media recruitment based on personal experiences are presented.

**Trial Registration:**

German Clinical Trials Register DRKS00021144; https://drks.de/search/en/trial/DRKS00021144

**International Registered Report Identifier (IRRID):**

RR2-10.1016/j.invent.2021.100410

## Introduction

### Background

Elevated levels of distress among patients with cancer [1-3] and lack of comprehensive psycho-oncological support, especially in the outpatient setting [4,5], have resulted in increased efforts to provide evidence-based psycho-oncological interventions (POIs). However, studies evaluating POIs face severe recruitment problems. In recent years, this situation has gained attention in the scientific literature, and a rising number of studies provide essential information on barriers to study participation. In a clinical correspondence, van Lankveld et al [6] called for a more extensive reporting about recruitment issues and negative experiences in psychosocial oncology research to share findings in the research community and to improve the feasibility of future studies by more realistic estimations of inclusion rates.

The reasons for low inclusion rates have been reported in previous studies and are manifold. In addition to recruitment barriers that occur in hospital-based recruitment among referring health care professionals (HCPs), such as limited time, increased workload, and prioritizing medical topics [7], studies identified barriers among patients with cancer that may prevent them from participating in POI studies. Limited interest in the intervention, no perceived symptom burden or need for a POI, scheduling difficulties, time commitment, and inappropriate timing of recruitment on the treatment trajectory (eg, too close to diagnosis) are among the reported barriers to study participation [8-11]. However, strict eligibility calculations and recruitment methods such as consecutive recruitment in hospitals may complicate patient enrollment, as they seem to lack practicability and feasibility in psycho-oncological research, often resulting in small sample sizes. For example, in a large multicenter randomized controlled trial (RCT) using consecutive screening for recruitment, only 25 (0.96%) of the 2608 approached patients with cancer were eligible and interested in participating in a psychological intervention, whereas the majority did not respond to the routine screening questionnaire, did not fulfill the inclusion criteria, already received treatment, or reported no need for treatment [11].

Web-based POIs may even face additional recruitment challenges compared with face-to-face POIs. Although the anonymity of web-based interventions may be regarded as an important benefit for people who feel more comfortable with anonymous help seeking (eg, because of the fear of stigmatization), other participants might wish for more personal contact to commit to the study. However, participants’ characteristics also explain their attitudes toward web-based interventions. A study investigating preferences for internet-based mental health interventions revealed that younger, female, and more educated participants were more likely to prefer web-based programs compared with face-to-face support [12]. In the context of POI research, the association of age and uptake of web-based interventions may be particularly important, as the likelihood of developing cancer increases with age, and up to now, older persons report increasing but still less internet use than younger persons [13].

To overcome the abovementioned recruitment challenges in POI research, it is proposed to combine hospital-based recruitment with participant self-referral [14]. It has been argued that self-referral might provide important benefits, such as increased ecological validity and thus successful implementation into practice [14]. In web-based research and mobile health research, self-referral through web-based study promotion (eg, via Facebook advertisements) is widely used as a recruitment method, either as a single method of recruitment or combined with traditional methods (eg, HCP referral, flyers, and newspapers). A systematic review by Lane et al [15] concluded that web-based recruitment methods may be promising in mobile health research, but more empirical evidence is needed on the effectiveness of web-based recruitment methods and participant retention, compared with traditional recruitment methods. The review emphasizes the benefits of web-based recruitment methods (such as wide reach, flexibility, and the potential to reach underserved populations) but also points to serious issues (eg, less investment and commitment of participants), which might limit the validity of research findings [15]. A scoping review reported inconclusive results regarding whether social media recruitment is more effective than traditional methods but found evidence that social media is the best method for recruiting hard-to-reach populations [16]. In the field of psycho-oncology, there is a rising number of studies that—either additionally or exclusively—use web-based and social media recruitment [17-21], underlining the effectiveness of social media and web-based recruitment, especially with regard to participant enrollment.

### Objectives

This secondary analysis is based on recruitment data gathered in the emotion-based psycho-oncological online self-help (*epos*) project. In this project, we developed and evaluated the web-based intervention *epos* that aimed at reducing psychological distress in people with cancer. Although it was not the explicit aim of the *epos* project to investigate and compare recruitment strategies, the study generated valuable data on the development and effectiveness of a recruitment strategy that combined traditional and web-based recruitment methods. The aim of this study is to provide results on the effectiveness of different recruitment strategies and to discuss the implications for improving response rates in psycho-oncological web-based intervention research. To achieve this, we will provide a comprehensive overview of our recruitment procedure, including the challenges we encountered, and explore how demographic and medical characteristics are linked to the recruitment method.

## Methods

### Study Design

The data reported in this study were collected from May 2020 to September 2021 within the scope of the *epos* project, in which we developed the web-based self-help program *epos* and evaluated its effectiveness in a monocentric RCT with a parallel group design. The RCT was registered at the German Clinical Trials Register (DRKS00021144).

Participants were randomly assigned to the intervention group (self-help program *epos*) or control group (treatment as usual+informational website) and completed 3 questionnaires (baseline, after intervention, and follow-up). In brief, participants in the intervention group had 10 weeks of access to *epos*, an intervention consisting of 1 introductory unit and 9 units related to specific psycho-oncological topics (eg, *talking about cancer* and s*trengthening the soul*). *Epos* is designed as a self-guided program, giving users the opportunity to navigate through the content in a self-determined manner. It is advisable to focus on 1 unit per week, although the time it takes to complete a unit can vary depending on how thoroughly users engage with the content. On average, users are expected to spend approximately 30 to 60 minutes on each unit. Detailed information on the study design of the RCT has been provided elsewhere [22].

For this study, only data on recruitment as well as demographic and medical data assessed in the baseline questionnaire were used. All procedures, including patient information, diagnostic self-assessment regarding eligibility, informed consent, and registration for the web-based intervention, were conducted via the internet. Direct contact with the research staff was offered via email or telephone if (potential) participants needed it.

### Ethical Considerations

All procedures were approved by the Ethics Committee of the Federal State of Rhineland-Palatinate (2019-14460) on July 26, 2019, and May 19, 2020. All study participants provided informed consent via an electronic form. To protect the privacy and confidentiality of the participants, study data underwent pseudonymization via assigned study ID numbers. No compensation was provided to the participants.

### Participants

Patients met the eligibility criteria if they satisfied the following conditions: (1) age ≥18 years, (2) diagnosed with any form of cancer, (3) currently received cancer treatment or in posttreatment care, (4) possessed adequate German language proficiency, and (5) had internet access. The exclusion criteria were severe mental or physical disabilities (eg, severe depression). Screening for inclusion and exclusion criteria was conducted via self-assessment of the participants.

### Procedures

Originally, the recruitment was planned to be conducted exclusively at the study center (University Medical Center of the Johannes Gutenberg University Mainz). The intended number of participants was determined based on our past experience, which considered the annual volume of oncological patients treated and the outcomes of routine distress screenings conducted in previous years. On the basis of 2018 and 2019 data, an average of 172 cancer patients received psycho-oncology care per month, of whom approximately 75% were undergoing curative treatment. Approximately 75% of these patients were assumed to meet inclusion criteria, resulting in an estimated number of 1354 eligible patients over the 14-month recruitment period. Thus, to achieve a sufficient number of participants of 325 patients, a 25% participation rate was required.

Owing to several reasons, the original recruitment strategy was revised and extended during the conceptual study phase. Recruitment experiences gained in the qualitative study, which was conducted between February and May 2019 during the intervention development phase [23], in which we aimed to assess the needs of patients with cancer by conducting interviews with 10 patients with cancer, showed that recruitment in the hospital was much lower than anticipated. The experience that many inpatient patients with cancer might be too burdened for study recruitment, implying that recruitment solely through direct contact by the HCP and in only 1 hospital would be less effective than expected, prompted us to revise the recruitment strategy. Targeted inquiries to several acute and rehabilitative hospitals resulted in 23 clinics that expressed an interest in supporting patient recruitment for the *epos* study free of charge. All hospitals received flyers of the *epos* study and were asked to display them in their clinics or even distribute them personally to potentially eligible and interested individuals to increase motivation.

**Figure 1:**
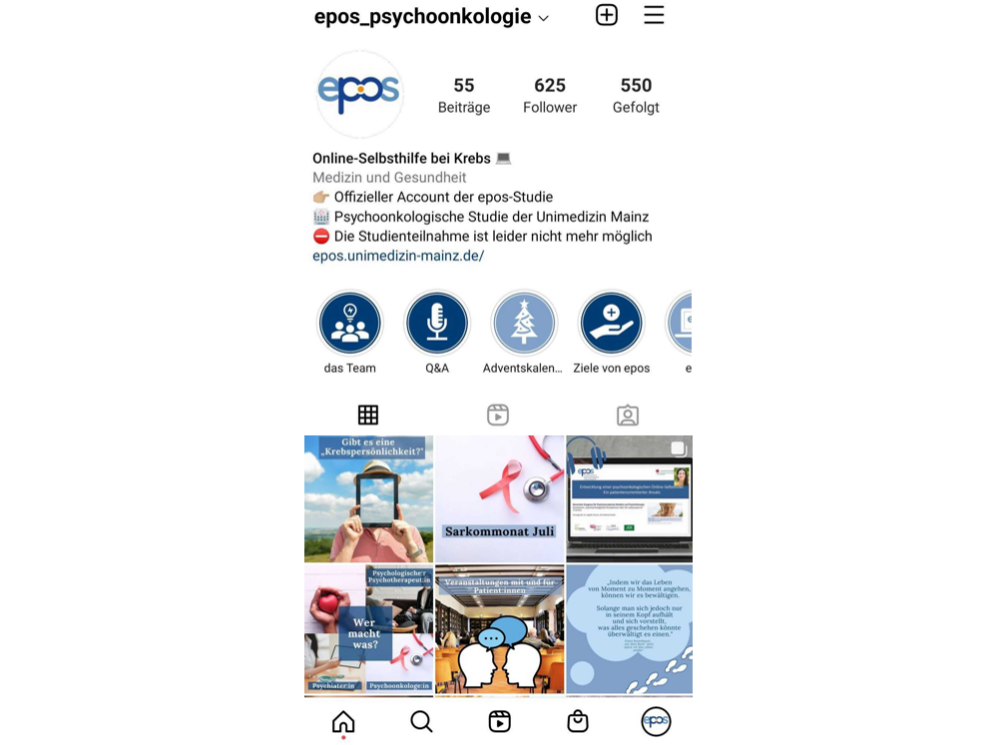
Instagram feed of the emotion-based psycho-oncological online self-help account.

The global COVID-19 pandemic severely affected medical and clinical routines beginning in February 2020 in Germany, including the recruitment for the RCT that began in May 2020 under lockdown conditions. Specifically, the research staff were not allowed to recruit participants in waiting areas or medical departments, as personal contacts should be as limited as possible to protect patients with cancer and medical staff. Hence, it was decided to extend the recruitment strategy and additionally integrate social media and web-based channels, as they experienced a strong demand owing to the pandemic in nearly every domain of life. Social media and web-based recruitment primarily involved recruitment activities through the Instagram and Facebook accounts of the *epos* study. The most prominent mechanism for recruiting via Instagram and Facebook are targeted advertisements (refer to the study by Arigo et al [24] for a valuable overview of the methodological and ethical considerations for using social media for health research). As web-based recruitment was not planned in the grant and study budget, costly advertisements (eg, on Facebook or Instagram) could not be afforded. Instead, we launched an *epos* Instagram account and a Facebook account, which we used for informing about the study and building a network within the community of survivors of cancer (Figure 1). Through own postings and reposts by influencer accounts with a wide reach, the Instagram account had 600 followers by the end of the recruitment phase. The Facebook account had substantially fewer followers, with 66 followers by the end of the recruitment phase. The posts on Instagram and Facebook were identical and were uploaded simultaneously. Social media posts mainly provided quotes or information, such as an introduction of the study team or psycho-educative topics such as the difference between a psychiatrist, a psychotherapist, and a psycho-oncologist, describing scientific methods for lay people (eg, describing an RCT) or informing about cancer awareness months. The main strategies for growing an Instagram community were reposts of our posts by cancer survival influencers or networks with a wide reach and generally increased social media activity (commenting and liking posts of relevant Instagram accounts and frequent posts, stories, and reels). Interactions with the community and networks of survivors of cancer have partly resulted in activities with wide reach, for example, an invitation to present *epos* at a conference for patients with breast cancer (*Mamma Mia!*) conducted on the internet via videoconferencing or an article in a breast cancer magazine.

Finally, a link to our study’s home page was presented on several websites, including self-help networks or federal associations for specific cancer types. To reach people with less digital activity, we published information about the *epos* study in classic media (eg, daily newspapers and local radio).

To describe or—where possible—even quantify recruitment strategies, we monitored flyer distribution to hospitals and other institutions as well as important social media activities conducted by the research staff.

For all participants in the *epos* study, registration followed the same procedure. Participants received the URL to the study home page (eg, via the flyer of the HCP or the Instagram account). On the study home page, participants were provided with the study information and registration link and could sign up for the study. After completion of the baseline questionnaire that was presented directly after registration, study participants were informed about group allocation and received access to *epos* or the content for the control group. More details on the registration procedure are provided elsewhere [22].

### Variables

#### Sociodemographic and Medical Characteristics

Patient-reported data presented in this study were collected via the internet using the baseline questionnaire. Demographic and medical characteristics included sex, age, marital status, education, nationality, internet use, cancer type, time since diagnosis, administered cancer treatment, metastases and recurrence, and treatment setting. The number of comorbidities was assessed by providing a list of 18 somatic or mental conditions and a free-text field for additional answers. Psychological distress was assessed using the 16-item Patient Health Questionnaire Anxiety and Depression Scale, a combined measure of depression and anxiety [25]. Participants indicated symptoms of depression and anxiety on a 4-point scale ranging from 0 (not at all) to 3 (nearly every day).

#### Paths of Recruitment and Study Referral

Two self-developed items assessed information on recruitment into the study. Recruitment path was measured by the item “How did you become aware of the study,” which provided 8 response options describing specific hospitals or (social) media methods as well as the response option “other” that could be specified in a free-text box. The data provided in the free-text box were recoded into the existing categories whenever possible. Repeated or similar answers in the free-text box were summarized as new response options for this item during data preparation. Furthermore, participants were asked whether they received an HCP recommendation for study participation (eg, from a psycho-oncologist, physician, or nurse) or if their study participation was based on self-referral. The response options were “yes, received an HCP recommendation” or “no, did not receive an HCP recommendation.”

#### Adherence

Adherence, including the frequency of log-ins and duration of time logged in (in min), was assessed using objective data collected within the software. As the study questionnaires were also included in the software that provided the intervention, the reported frequency and log-in durations also included the questionnaire sessions, which represents a serious limitation for the validity of these data. To counteract this systematic bias, we also measured active engagement with the intervention content, which was operationalized by an activity score representing the proportion of completed interactive tasks within the content units. These interactions included, for example, filling out a free-text field or answering a multiradio question. In addition to treatment adherence measures, we tracked the number of completed study questionnaires intended to be completed at baseline, after the intervention, and at the 3-month follow-up, which allowed us to draw conclusions about trial adherence.

### Statistical Analyses

Descriptive analyses were used to quantify recruitment strategies and determine the demographic and medical characteristics of the participants. Chi-square analyses and unpaired 2-tailed *t* tests were used to compare groups based on the recruitment method. Logistic regression analysis estimating odds ratios with 95% CIs was performed to determine the factors associated with self-referral. Self-referral was dummy coded as 0=not self-referred and 1=self-referred. Statistical analyses were conducted using SAS software (version 9.4; SAS Institute). We defined the level of significance at *P*<.05; we additionally report larger effects (*P*<.01 and *P*<.001). Because of the exploratory nature of this study, we did not perform alpha adjustment.

## Results

### Participant Characteristics

A total of 327 participants provided informed consent and were randomized into the RCT. After excluding 19 participants owing to withdrawals and incomplete data in the baseline questionnaire, the final population consisted of 308 participants. As 4 participants did not provide information on the recruiting strategy, data from 304 participants were used to analyze the population based on recruitment methods. The sociodemographic characteristics of the participants are shown in Table 1, and their medical characteristics are presented in Table 2. Briefly, the study population consisted of 84.7% (249/294) female participants with a mean age of 50.8 (SD 10.9) years. Significant group differences between traditionally and web-recruited participants were identified for sex, age, internet use, study referral, and treatment status.

**Table 1:** Sociodemographic characteristics of the study participants (N=304).

Characteristics		Total sample	Traditional recruitment^a^ (n=153)	Web-based recruitment^b^ (n=151)	*P* value	
**Sex, n (%)**						*<.001* ^c^
	Female	249^d^ (84.7)	114 (77.6)	135 (91.8)		
	Male	45 (15.3)	33 (22.4)	12 (8.2)		
Age (years), mean (SD; range)		50.8 (10.9; 24-83)	52.5 (10.1; 30-83)	49.0 (11.4; 24-78)	*.005*	
**Marital status, n (%)**						.72
	Single	29 (9.6)	14 (9.3)	15 (10)		
	In a relationship or married	233 (77.4)	115 (76.2)	118 (78.7)		
	Divorced, separated, or widowed	39 (13)	22 (14.6)	17 (11.3)		
**Education, n (%)**						.89
	No degree or lower secondary education diploma	16 (5.3)	9 (5.9)	7 (4.6)		
	General secondary education diploma	56 (18.4)	27 (17.6)	29 (19.2)		
	Diploma qualifying for university	228 (75)	115 (75.2)	113 (74.8)		
	Other degree	4 (1.3)	2 (1.3)	2 (1.3)		
German nationality, n (%)		289 (96)	147 (97.4)	142 (94.7)	.23	
**Private internet use (min), mean (SD)**						
	On weekdays	106.7 (96.8)	91.8 (83.7)	121.7 (106.7)	*.007*	
	On weekends	109.8 (104.8)	95.2 (89.0)	124.3 (117.0)	*.02*	
**Study referral, n (%)**						*<.001*
	Self-referred	236 (79.7)	91 (61.5)	145 (98)		
	Referred by a health care professional	60 (20.3)	57 (38.5)	3 (2)		

^a^Traditional recruitment includes all offline recruitment activities, that is, recruitment through health care professionals in hospitals and medical practices, cancer counseling centers, self-help networks, and print media.

^b^Web-based recruitment includes all web-based recruitment strategies, that is, social media (Instagram and Facebook) and study promotion on websites.

^c^Italicized values represent significant differences between web-based and traditional recruitment

^d^Numbers may not add up to 304 owing to missing data.

**Table 2:** Medical characteristics of the study participants (N=304).

Characteristics		Total sample	Traditional recruitment^a^ (n=153)	Web-based recruitment^b^ (n=151)	*P* value	
**Cancer type, n (%)**						.41
	Breast	178^c^ (58.6)	82 (53.6)	96 (63.6)		
	Hematologic	29 (9.5)	15 (9.8)	14 (9.3)		
	Gynecologic	25 (8.2)	11 (7.2)	14 (9.3)		
	Skin	13 (4.3)	10 (6.5)	3 (2)		
	Colon	11 (3.6)	5 (3.3)	6 (4)		
	Head and neck and thyroid	10 (3.3)	4 (2.6)	6 (4)		
	Prostate	8 (2.6)	7 (4.6)	1 (0.7)		
	Other^d^	30 (9.9)	19 (12.4)	11 (7.3)		
Time since diagnosis (in weeks), mean (SD)		74.0 (118.5)	82.9 (145.0)	64.9 (82.9)	.18	
Metastases, n (%)		93 (31.4)	45 (30)	48 (32.9)	.59	
Cancer recurrence, n (%)		31 (10.6)	18 (12.4)	13 (8.8)	.31	
Ongoing acute treatment^e^, n (%)		170 (55.9)	77 (50.3)	93 (61.6)	*.048* ^f^	
**Treatment setting, n (%)**						.39
	Inpatient	8 (2.7)	4 (2.7)	4 (2.7)		
	Outpatient	163 (54.9)	76 (51)	87 (58.8)		
	After care	126 (42.4)	69 (46.3)	57 (38.5)		
Number of somatic or mental comorbidities, mean (SD; range)		1.7 (1.8; 0-13)	1.6 (1.6; 0-7)	1.8 (2.0; 0-13)	.51	
Psychological distress^g^, mean (SD; range)		17.8 (8.3; 0-44)	17.2 (8.0; 0-38)	18.5 (8.6; 3-44)	.20	

^a^Traditional recruitment includes all offline recruitment activities, that is, recruitment through health care professionals in hospitals and medical practices, cancer counseling centers, self-help networks, and print media.

^b^Web-based recruitment includes all web-based recruitment strategies, that is, social media (Instagram and Facebook) and study promotion on websites.

^c^Numbers may not add up to 304 owing to missing data.

^d^Tumor sites are as follows: brain, liver, pancreatic, and testicular or penile: 0%; kidney, bladder, and stomach: 1%; and lung and soft tissue: 2%.

^e^Including chemotherapy, radiation, immune therapy, and hormone therapy.

^f^Italicized values represent significant differences between web-based and traditional recruitment methods.

^g^Assessed with the Patient Health Questionnaire Anxiety and Depression Scale (combined measure of anxiety and depressive symptoms); a higher total score indicates higher psychological distress.

### Flyer Distribution

**Figure 2:**
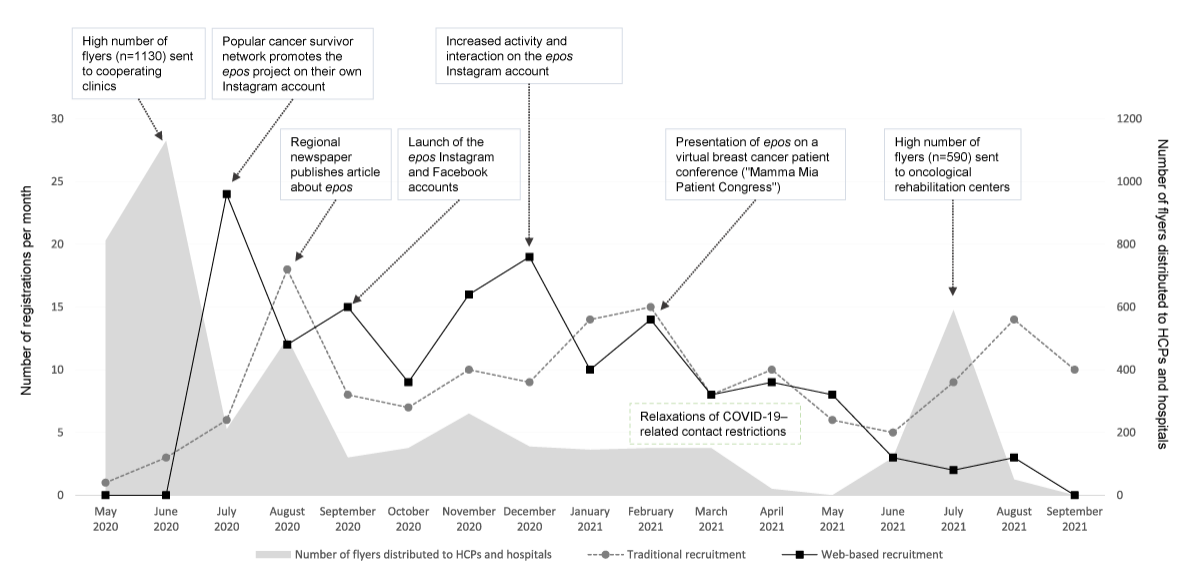
Important recruitment activities and registration numbers over time. HCP: health care professional.

Figure 2 provides an overview of important traditional and web-based recruitment activities (eg, flyer distributions and social media activities) and registration numbers during the recruitment period. Traditional recruitment includes all offline recruitment activities, that is, recruitment through HCPs in hospitals and medical practices, cancer counseling centers, self-help networks, and print media. Web-based recruitment includes all web-based recruitment strategies, that is, social media (Instagram and Facebook) and study promotion on websites.

In Table 3, we report the number of cooperating institutions and flyers distributed during the 17-month recruitment period. A total of 4561 study flyers were provided to clinics, medical practices, and other institutions during the recruitment phase.

**Table 3:** Distribution of flyers (N=4561) in cooperating hospitals, medical practices, and institutions (N=78).

Institutions	Hospitals, medical practices, and institutions, n (%)	Flyers provided^a^ n (%)
Internal recruitment—study center Mainz^b^	1 (1.3)	1371 (30.1)
External recruitment^c^	70 (89.7)	2910 (63.8)
Other (eg, cancer counseling centers and self-help networks)	7 (9)	280 (6.1)

^a^Number of flyers provided to the hospital, medical practice, or institution: this number does not include any information on the number of flyers that were eventually handed out to patients with cancer. Owing to reasons of clinical practicability, the total number of flyers that reached patients with cancer could not be assessed.

^b^Includes all departments at the University Medical Center of the Johannes Gutenberg–University Mainz relevant for cancer treatment as well as active health care professional recruitment and flyer distribution for self-referral at information desks, etc.

^c^External recruitment includes acute hospitals, specialized oncological practices, and oncological rehabilitation hospitals.

### Paths of Recruitment and Study Referral

The absolute numbers and percentages for paths of recruitment are displayed in Table 4. Most participants (151/304, 49.7%) became aware of the study through web-based activities, whereas 34.9% (106/304) were recruited in hospitals, medical institutions, and practices: 17.4% (53/304) were treated in the Mainz study center and 17.4% (53/304) were treated in cooperating hospitals and medical practices.

A total of 296 participants provided data on whether they registered as self-referred or owing to an HCP recommendation. The majority of participants (236/296, 79.7%) registered as self-referred. The associated demographic and medical characteristics for self-referral are displayed in Table 5. Younger age, currently receiving cancer treatment, and nonuse of psycho-oncological offers were identified as significant variables associated with self-referral.

**Table 4:** Participants’ reported paths of recruitment (n=304).

Paths of recruitment	Participants, n^a^ (%)
Web-based	151 (49.7)
Internal recruitment—study center Mainz	53 (17.4)
External recruitment^b^	53 (17.4)
Print media	30 (9.9)
Through friends or family members	13 (4.3)
Self-help network and cancer counseling center	7 (2.3)
Other	5 (1.6)

^a^The total number of responses given regarding paths of recruitment exceeds 304, as multiple responses were allowed.

^b^External recruitment includes acute hospitals, specialized oncological practices, and oncological rehabilitation hospitals.

**Table 5:** Demographic and medical predictors for self-referred registration^a^ (n=236).

Variable		Odds ratio (95% CI)	*P* value
**Sex**			
	Female	Reference	N/A^b^
	Male	0.48 (0.21-1.08)	.08
**Age (years)**			
	Per 1 additional year	0.95 (0.92-0.99)	*.006* ^c^
**Time since diagnosis**			
	Per 1 additional week since diagnosis	1.00 (1.00-1.00)	.58
**Ongoing cancer treatment^d^**			
	No	Reference	N/A
	Yes	1.98 (1.02-3.84)	*.04*
**Prior use of psycho-oncological offer**			
	No	Reference	N/A
	Yes	0.23 (0.10-0.52)	*<.001*
**Psychological distress^e^**			
	Minimal (0-9)	Reference	N/A
	Mild (10-19)	1.16 (0.46-2.91)	.76
	Moderate (20-29)	1.45 (0.55-3.80)	.45
	Severe (30-48)	0.68 (0.20-2.38)	.55
**Education level^f^**			
	Lower education	Reference	N/A
	Higher education	1.11 (0.52-2.42)	.78

^a^Participants who indicated that they had registered for the study without a health care professional recommendation.

^b^N/A: not applicable.

^c^Italicized values represent significant *P* values.

^d^Including chemotherapy, radiation, immune therapy, and hormone therapy.

^e^Assessed with the Patient Health Questionnaire Anxiety and Depression Scale (combined measure of anxiety and depressive symptoms).

^f^Lower education: no degree, lower and general secondary education diploma; higher education: diploma qualifying for university.

### Adherence

For the 155 participants who were randomized into the intervention group and therefore had access to the web-based self-help program, we calculated the number of log-ins, mean duration of log-in time, and activity within the web-based self-help program. Mean frequency of log-ins was 8.26 (SD 7.22) for traditional and 6.47 (SD 4.98) for web, mean duration of log-ins (in min) was 353.56 (SD 553.79) and 227.95 (SD 239.28), respectively, and mean proportion of completed interactive tasks within the program (in percentage) was 31.4% (SD 34.8) and 23.8% (SD 29.2), respectively. There were no significant differences in the frequency of log-ins, duration of log-ins, and activity within the program between the participants who were recruited traditionally and those who became aware of the study through a web-based recruitment channel.

In terms of trial adherence, 68% (52/77) of the traditionally recruited participants completed all 3 study questionnaires. In comparison, the completion rate of all 3 questionnaires was slightly lower among the participants recruited web-based (47/78, 60%). However, this difference was not statistically significant, indicating that the groups did not differ in trial adherence.

## Discussion

### Principal Findings

The aim of the study was to outline the development and effectiveness of the recruitment strategy of an RCT evaluating the newly developed psycho-oncological web-based intervention *epos*. The recruitment of participants for the RCT had to be adapted because of the challenges previously reported in POI research and the impact of the COVID-19 pandemic. At the start of the recruitment phase, which initially relied on the distribution of flyers in hospitals and health care provider referrals, it did not meet the anticipated and necessary participant numbers. However, a significant increase was only achieved through social media activities. The final sample was not representative, with female participants, younger age, and higher education being overrepresented. Over the course of the recruitment phase, web-based recruitment was the most successful recruitment strategy with regard to participant registration numbers, followed by recruitment in the study center Mainz. Participants who self-referred to the study were found to be more likely to be younger, undergoing current cancer treatment, and not have used psycho-oncological offers in the past compared with participants who were referred to the study by an HCP.

### Comparison With Prior Work

In general, the recruited sample was not representative of several sociodemographic characteristics, especially with regard to the overrepresentation of female participants, younger age, and higher education. Female sex has been associated with the perceived need for or uptake of psycho-oncological face-to-face offers in previous German studies [4,5,26], suggesting that the self-selection bias revealed in this study is not just a matter of the web-based format and the recruitment procedure; rather, it is a well-known issue emphasizing that male patients with cancer are less likely to perceive the need for or make use of POIs. Similarly, the overrepresentation of higher education is in line with previous findings that suggest that the uptake of psycho-oncological support is associated with higher education [4,5]. Among the web-recruited study participants, the proportion of female participants was even higher compared with traditionally recruited participants, the mean age was lower, private internet use was higher, and the proportion of participants who were currently under treatment was higher. The remaining demographic and medical characteristics revealed no significant differences, suggesting that the web-based and traditionally recruited participants were comparable in terms of these characteristics. This finding is partly in line with a systematic review that found populations recruited via Facebook to be comparable with control populations, except for an overrepresentation of female participants and younger age groups [27]. Younger age among web-recruited participants might reflect the age demographics of Instagram users, with more than half of the global Instagram population being aged ≤34 years [28]. In terms of intervention and trial adherence, we observed no significant differences between participants using the web-based and traditional recruitment methods. Thus, our data do not support the assumption that web-recruited participants were less committed to the study than traditionally recruited participants, which has been reported as a possible limitation to validity elsewhere [15].

Although the overrepresentation of female participants is a frequently described finding in mental health research in general and also specifically in web-based POI research [19,20,29-32], it is unfortunate that more male participants could not be recruited for the study. Considering this potential self-selection bias, different measures were taken in the early phase of the study conception to gain the attention of male participants for *epos*. Special efforts were made in designing *epos* in a way that equally represented prototypes of male and female patients with cancer, not only in the written content but also visually with pictures showing prototypes of male patients with cancer. In the recruitment phase, we designed an additional version of the recruitment flyer with pictures of male patients and slightly different wording and icons (more technical and less emotion-based language). However, these specific measures did not come close to balancing out the other activities that targeted significantly more female participants (eg, the presentation of *epos* at a digital congress for survivors of breast cancer). As the followers of the *epos* Instagram account were predominantly female, our social media promotion activities mainly reached female participants. The aim of reaching out to male participants, who might be hesitant to make use of face-to-face offers because of the fear of stigma, was not achieved in this study. This suggests that a POI designed for all genders and cancer types (as *epos* was) might be too nonspecific to attract male and female participants equally and that recruitment might be more effective if the POI is designed specifically for the male population (as, for instance, in the study by Wootten et al [33]).

Most participants (236/296, 79.7%) registered as self-referred without an HCP recommendation, despite great efforts in face-to-face recruitment via the HCP. Self-referral was predicted by younger age, which might be associated with the web-based recruitment strategy, as web-recruited participants were younger and web-based recruitment is analogous to self-referred recruitment. To our knowledge, evidence on the demographic or medical predictors of self-referral into web-based intervention studies is scarce. A study investigating the success of different recruitment methods for a mobile internet intervention RCT with postpartum mothers found that HCP-referred mothers had higher levels of risk factors compared with self-referred mothers, concluding that the recruiting staff might have prioritized approaching female participants who were perceived as most vulnerable [34]. Our findings revealed no significant differences in psychological distress between self-referred and HCP-referred participants, suggesting that elevated distress levels did not play a central role in recruitment via HCP referral. Further research is needed to understand what characteristics play a role in how patients with cancer find their way into web-based intervention research.

Recruiting exclusively at the study center Mainz was less effective than originally estimated and did not result in the planned inclusion rates. Thus, the gradual expansion of the recruitment strategy to other hospitals and medical practices was necessary. As a result, the inclusion rates of participants recruited in the medical or clinical setting increased significantly but still did not exceed the inclusion rates that were achieved through web-based recruitment. Despite the comparably high effort, we conclude that traditional recruitment, especially hospital-based recruitment and HCP referral, is still crucial. Although both groups, traditionally and web-recruited participants, were not representative of several sociodemographic characteristics, we observed a higher proportion of male participants in the traditional recruitment setting (33/147, 22.4%) compared with the web-based recruitment setting (12/147, 8.2%), which is an important finding with regard to low participation in male participants. This finding indicates that diversified recruitment strategies may be suitable for successful recruitment into clinical trials in POI research, as suggested in previous studies [18,35].

### Implications for Social Media Recruitment

The well-known challenges in hospital-based recruitment combined with the severe recruitment restrictions in hospitals owing to the COVID-19 pandemic have led us to engage in web-based recruitment activities. With web-based recruitment as a successful recruitment strategy, our results are in line with previous studies that emphasize the effectiveness of social media for recruiting patients with cancer into POI studies [18-20]. The 2 peaks in participant enrollment (June 2020 and February 2021) were temporally related to relevant social media activity. However, we noticed that recruitment via social media was not at a constant high level, as the number of followers did not increase steadily. We experienced a ceiling effect caused by a low turnover among followers, as already described elsewhere [19]. Although initial social media posts have led to a notable increase in participant numbers, subsequent actions had a smaller impact on registration numbers, indicating a serious limitation of social media recruitment. Another difficulty in social media recruitment, as conducted in this study, is creating an account that can compete with the fast-paced social media environment. As we did not use paid advertisements, we had to increase the account’s visibility through regular content and interaction with other Instagram accounts (eg, liking and commenting on posts of other accounts and answering comments under our posts), always taking into account the methodological and ethical considerations for the use of social media in health research, which is associated with specific challenges [24]. From our personal experience in recruiting on social media, several factors appeared to be crucial in the development of posts and maintenance of the *epos* Instagram account, leading us to the following recommendations:

- Do not underestimate the workload behind a professional social media account. Creating new content (eg, posts and stories) is time intensive, including the design of the post, selection of a picture, and writing of appropriate captions. Unlike paid advertisements, building a social media community involves intensive community interaction, for example, with followers or other professional accounts. These interactions might happen to be outside regular working hours, in the evening, or on weekends.
- Before launching a study account, we recommend taking some time to observe and become familiar with the platform. As the social media environment is an emerging, but still not the usual, terrain for researchers, observing social media communities will help understand the code of conduct in the targeted population. Bringing someone with more social media experience to your team, for example, research assistants, can be a huge advantage.
- Be professional, authentic, and clear in the description of your study account to ensure that followers understand what they can or cannot expect and especially not expect from following your account. Especially in the often mentally burdened community of survivors of cancer, followers may ask for psychological help or counseling. Be prepared for such requests, and refer them to appropriate supportive services, for example, the cancer counseling centers. Furthermore, do not try to disguise the aim of the account, which is to promote the study (and maybe inform about the research activities), as it is inappropriate to mislead followers with false promises.
- As every Instagram user can check which other accounts are being followed by the research project’s account, ensure to carefully decide whom to follow and avoid following untrustworthy accounts (eg, pseudomedical accounts). Rather, follow other HCPs who have trustworthy content.
- Encourage activities and comments under new posts but try to avoid comments that might lead to critical situations, for example, avoid asking questions that might invite participants of the study to report their experiences with the web-based intervention, as study-related comments could bias other participants.
- Try to avoid topics that could lead to negative (participant) responses in the comments of a post, as this might severely impact the project’s reputation. Carefully check (ideally by multiple people) the wording of your posts, the pictures that are used for the post, and the timing of new content.
- Carefully review the content you post on the study account for its potential impact on the psychological variables considered in the study. As it is impossible to control who views the account, do not post content that could potentially bias the study results.

### Limitations and Strengths

The results of this study need to be interpreted in light of several limitations. First, it was not possible to calculate response rates or provide reasons for nonparticipation owing to the study design and recruitment strategy. To increase the commitment of HCPs and practicability in the complex and time-constrained hospital setting, we did not ask the HCPs to provide a documentation of distributed flyers or reasons for nonparticipation expressed by patients. The number of distributed flyers reported in this study refers to flyers that were handed out to the HCPs or were sent to hospitals or other cooperating institutions. It is not possible to draw any conclusions on how many of these flyers finally reached patients with cancer. Thus, it remains unclear whether the low response rate in hospital-based recruitment is owing to a lack of interest in study participation on behalf of patients or rather owing to lower recruitment activities than expected on the side of the HCPs (eg, owing to limited time). Second, it is not possible to report response rates for web-based recruitment strategies, as the link to our study home page was widely distributed and we did not use paid advertisements that provide statistics on the number of clicks. Third, following an adapted and combined strategy instead of a strict recruitment strategy could be seen as a methodological limitation. On the one hand, we acknowledge that our study lacks the benefits of consecutive recruitment; on the other hand, the described recruitment procedure might help overcome recruitment barriers in psycho-oncology and those related to the COVID-19 pandemic. Fourth, the reduced interpretability of adherence data is a further limitation. As mentioned in the section describing the adherence variables, the validity of objective adherence measures was limited, as these data do not only include adherence but also log-in data that were collected while participants completed the questionnaires. However, we sought to counteract this limitation by calculating an adherence measure that is based on the participants’ activity within the intervention. Finally, although we developed strategies to recruit a representative population (eg, male representation in the intervention and recruitment material and using traditional and social media recruitment), the final sample was not representative of sex, age, and education levels.

The flexibility of the recruitment procedure represents a strength. COVID-19–related recruitment problems that came on top of preexisting challenges in psycho-oncological research were countered by strengthening cooperation with other hospitals, networks, and institutions and by using social media methods. By combining HCP referral and self-referral, we fulfilled the number of intended participants, and the distribution enabled comparison of patient characteristics in subgroup analyses. The recruitment strategies described in this study represent an efficient method to create a wide reach.

### Conclusions

This study outlined the development and effectiveness of a diversified recruitment strategy for a clinical trial evaluating an unguided psycho-oncological web-based intervention. In addition, implications and recommendations for social media recruitment based on personal experiences were presented. Although traditional recruitment did not result in the planned inclusion rates, social media recruitment provided a substantial increase in participant numbers. Although the population was not representative of several sociodemographic characteristics, we conclude that combining traditional recruitment in hospitals with web-based and social media recruitment is a feasible and effective method to overcome recruitment barriers. Given that almost half of the participants were recruited web-based, we recommend considering web-based recruitment as a viable option in POI research; this approach can enhance practicality and ecological validity. However, evidence on the benefits and pitfalls of social media recruitment in POI research is limited. Future studies may provide further evidence on how best to combine traditional and web-based recruitment in terms of increasing inclusion rates while maintaining validity to ensure quality research.

## Abbreviations

epos: emotion-based psycho-oncological online self-help
HCP: health care professional
POI: psycho-oncological intervention
RCT: randomized controlled trial
